# Supplementary material for: Identification of photoperiod-induced specific miRNAs in the adrenal glands of Sunite sheep (Ovis aries)
Source: Front Vet Sci. 2022 Jul 22;9:888207. doi: 10.3389/fvets.2022.888207 (PMC9354845; doi:10.3389/fvets.2022.888207)
Supplement: Supplementary file 1 [file Data_Sheet_1.ZIP › Supplementary file/miRDeep2 file.zip!/miRDeep2 file/Novel_Structure/SPLP42A2/Novel_216.pdf]

Provisional ID	:	NC_019478.2_37268
Score total	:	1.9
Score for star read(s)	:	-1.3
Score for read counts	:	0
Score for mfe	:	1.6
Score for randfold	:	1.6
Score for cons. seed	:	
Total read count	:	444
Mature read count	:	444
Loop read count	:	0
Star read count	:	0

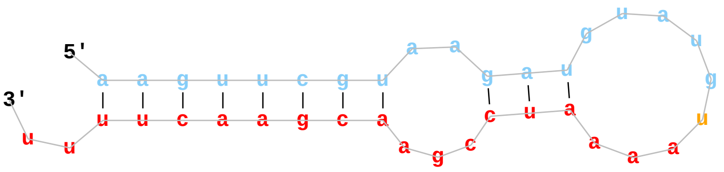

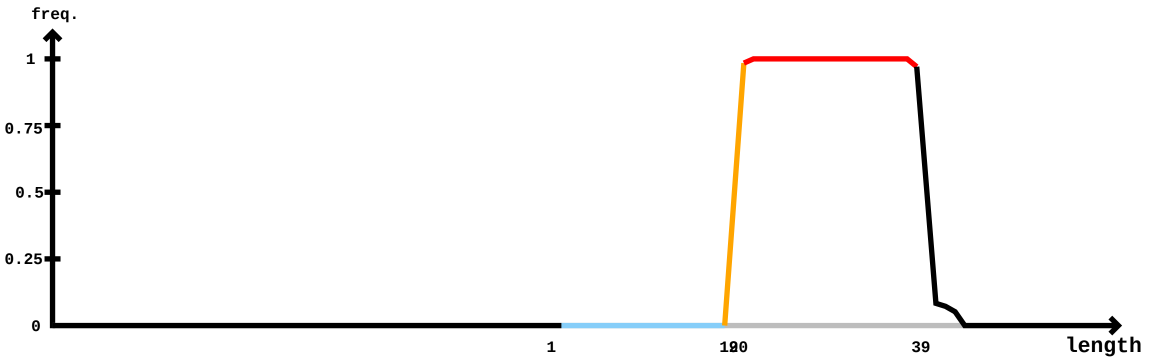

Star Mature

```
5'- gcagaggaaggaaggagggggugucugagaggguauugggcuugggccagaaaguucguaagauguauguaaaauccgaacgaacuuuuugggcccacccaguauuuca   -3'   exp
    .((((.(..............).))))...((((((((((..(((((((((((((((((..(((........)))...))))))))))).))))))))))))))))..   reads   mm   sample
    ....................................................................uaaaauccgaacgaacuuuuug..................   1       0    seq
    .....................................................................aaaauccgaacgaacuuu.....................   13      0    seq
    .....................................................................aaaauccgaacgaacuuuu....................   387     0    seq
    .....................................................................aaaauccgaacAaacuuuuu...................   3       1    seq
    .....................................................................aaaauccAaacgaacuuuuu...................   4       1    seq
    .....................................................................aaaauccAaacgaacuuuuug..................   1       1    seq
    .....................................................................aaaauccgaacAaacuuuuug..................   3       1    seq
    .....................................................................aaaauccgaacAaacuuuuugg.................   1       1    seq
    .....................................................................aaaaucUgaacgaacuuuuugg.................   2       1    seq
    .....................................................................aaaauccgaacgaacuuuuuggg................   22      0    seq
    ......................................................................aaauccgaacgaacuuuuugg.................   6       0    seq
    ......................................................................aaauccgaacgaacuuuuuggg................   1       0    seq
```
